# Divergent roles of the acetyl-CoA synthetases RkACS1 and RkACS2 in carotenoid and lipid biosynthesis in *Rhodosporidium kratochvilovae*

**DOI:** 10.1007/s00253-025-13534-x

**Published:** 2025-06-07

**Authors:** Meixia He, Xiaoxia Yang, Chao Xiong, Yuxuan Gan, Hongjun Ma, Jingwen Qiu, Yuan Chen, Qi Zhang

**Affiliations:** Faculty of Life Science and Technology, Kunming University of Science and Technology, Kunming, 650500 China

**Keywords:** *Rhodosporidium kratochvilovae*, Acetyl-CoA, Acetyl-CoA synthetase, Carotenoids, Lipids, Fatty acids

## Abstract

**Abstract:**

Red yeasts demonstrate considerable potential in industrial and biotechnological applications, particularly in the biosynthesis of carotenoids and lipids, which are valuable secondary metabolites with a wide range of applications. In the oleaginous red yeast *Rhodosporidium kratochvilovae* YM25235, the acetyl-CoA synthetases RkACS1 and RkACS2 play critical roles in converting acetate into acetyl-CoA, a key precursor for the synthesis of various metabolites, including carotenoids and lipids. This study explores the physiological functions and metabolic regulation of RkACS1 and RkACS2, revealing distinct roles for these isoenzymes in metabolic processes. RkACS1 is essential for utilizing non-fermentable carbon sources such as acetate, ethanol, and glycerol, exhibiting high affinity for acetate and being activated by acetate while inhibited by glucose. Additionally, RkACS1 is involved in carotenoid biosynthesis. In contrast, RkACS2, while not specific to particular carbon sources, is primarily involved in lipid and fatty acid synthesis. It also influences gene expression through histone acetylation in the nucleus. Notably, these two isoenzymes exhibit functional redundancy and mutual regulation. These findings provide valuable insights into the metabolic regulation of acetyl-CoA synthesis, offering a foundation for engineering strategies aimed at optimizing secondary metabolite production in oleaginous red yeasts.

**Key points:**

• *RkACS1 is related to carotenoid biosynthesis and essential for non-fermentable carbon sources*

• *RkACS2 supports lipid and fatty acid biosynthesis and regulates histone acetylation in the nucleus*

• *Functional redundancy and mutual regulation exist between RkACS1 and RkACS2 isoenzymes*

**Supplementary Information:**

The online version contains supplementary material available at 10.1007/s00253-025-13534-x.

## Introduction

*Rhodosporidium*, basidiomycete yeast belonging to the order *Sporidiobolales*, biosynthesizes antioxidant carotenoids primarily consisting of β-carotene, γ-carotene, torulene, and torularhodin (Buzzini et al. 2007). Moreover, it is also a promising microbial lipid producer (Castañeda et al. 2024; Mota et al. 2022; Qi et al. 2020), synthesizing triacylglycerols (TAGs) enriched with saturated and monounsaturated fatty acids (Szotkowski et al. 2021). The biological features of *Rhodosporidium* in carotenoid and lipid biosynthesis reflect its potential application value in the fields of industry and biotechnology (Mannazzu et al. 2015; Szotkowski et al. 2021), such as biodiesel production (Jiru et al. 2017), carotenoid extraction (Szotkowski et al. 2021), and the degradation of waste materials (e.g. lignocellulose) (Patel et al. 2015). *Rhodosporidium kratochvilovae* YM25235 strain was isolated from the high-altitude Chenghai Lake in Yunnan, China, where the temperature is low for most of the year and nutrients are relatively limited. Our previous studies have elucidated the adaptation mechanisms of this strain to low temperature and glucose starvation, revealing that its adaptability to environmental stresses is closely related to the biosynthesis of carotenoids and polyunsaturated fatty acid (PUFA) biosynthesis (Guo et al. 2021, 2022; He et al. 2019, 2023; Wang et al. 2017). Among these, the carotenoid biosynthesis capacity directly reflects the antioxidant capability of the cells (Zhang et al. 2022).

Carotenoids are potent antioxidants that protect cells from oxidative damage, stabilize cell membranes, and scavenge free radicals (Widomska and Subczynski 2019). They exhibit multiple health benefits, including anti-inflammatory effects, vision protection, skin protection, immune enhancement, and prevention of cardiovascular diseases and cancer (Riccioni 2009). Carotenoids are closely associated with lipids, as liposomes and lipid droplets serve as their storage and transport carriers, maintaining intracellular carotenoid stability within cells (Gao et al. 2017). Fatty acids, essential lipid components, preserve membrane integrity, regulate cell signaling, and provide antioxidant defense (Henry et al. 2002). PUFAs, especially ω−3 PUFAs such as α-linolenic acid (ALA, C18:3), eicosapentaenoic acid (EPA, C20:5), and docosahexaenoic acid (DHA, C22:6), are crucial for human health, contributing to cardiovascular disease prevention, inflammation reduction, and tumor growth inhibition (Liang et al. 2008; Wang et al. 2015). Despite the significant health value of carotenoids and fatty acids, efficient industrial production remains challenging (Power et al. 2022).

The biosynthesis of various natural products, including carotenoids, fatty acids, and lipids, relies on Acetyl coenzyme A (acetyl-CoA) as a key precursor (Power et al. 2022). Acetyl-CoA is involved in nearly 200 cellular reactions (Hynes and Murray 2010) and plays a central role in energy metabolism and biosynthesis (Ali et al. 2024; Strijbis and Distel 2010). In yeast, acetyl-CoA is primarily derived from carbohydrate, lipid, and amino acid catabolism (Chen et al. 2012). In *Saccharomyces cerevisiae*, acetyl-CoA synthesis occurs in multiple cellular compartments (Zhang et al. 2021). Mitochondrial acetyl-CoA, produced via the pyruvate dehydrogenase (PDH) complex, enters the tricarboxylic acid (TCA) cycle for energy generation (Lee et al. 2020; Zhang et al. 2021). Peroxisomal β-oxidation (Hiltunen et al. 2003) and the cytoplasmic PDH bypass pathway (Tang et al. 2013) also contribute to acetyl-CoA production. In the nucleoplasm, acetate is converted into acetyl-CoA to provide acetyl groups for histone acetylation, regulating chromatin structure and gene expression (Takahashi et al. 2006). Thus, acetyl-CoA integrates metabolic pathways across cellular compartments and serves as a key regulatory molecule in cellular processes (Zhang et al. 2021).

Cytosolic acetyl-CoA serves as a substrate for synthesizing various derivatives (Zhang et al. 2021) and is primarily supplied through the PDH bypass pathway, where pyruvate is converted into acetate by pyruvate decarboxylase (PDC), and further into acetyl-CoA by acetyl-CoA synthetase (ACS), the key enzyme in this pathway (Tang et al. 2013). ACS is the only enzyme in yeast capable of catalyzing this conversion (Kozak et al. 2014), and isoenzymes, ACS1 and ACS2, are essential for cell survival, as their dual deletion is lethal (Pronk et al. 1994; Van den Berg and Steensma 1995). *ACS1* gene encodes an aerobic enzyme that is inhibited by glucose and activated by acetate, while *ACS2*, encodes a non-aerobic enzyme required for growth on glucose (Liu et al. 2016; van den Berg et al. 1996). The expression of these isoenzymes is regulated by specific growth conditions. Initially, ACS1 was believed to be confined to mitochondria and peroxisomes (De Virgilio et al. 1992), but later study revealed its presence in the nucleus and cytoplasm as well (Huh et al. 2003). ACS2 is predominantly located in the nucleus, though it may also reside in the endoplasmic reticulum (ER) (Falcón et al. 2010). ACS2 influences global transcription by modulating histone acetylation within the nucleus (Takahashi et al. 2006). Research has demonstrated that ACS2 influences fatty acid biosynthesis genes via transcription factors INO2 and INO4 (Hiesinger et al. 1997). Although both isoenzymes catalyze acetate activation, their differing regulatory mechanisms, expression patterns, and localization drive distinct metabolic roles (van den Berg et al. 1996).

Our previous study revealed that glucose starvation significantly enhances the PDH bypass pathway, with up-regulation of *ACS* genes (He et al. 2023). Overexpressing *ACS1* and *ACS2* notably increases intracellular acetyl-CoA levels, but their interplay and role in secondary metabolite synthesis remain unclear. This study investigates the physiological functions of RkACS1 and RkACS2 in *R. kratochvilovae* YM25235, focusing on their impact on acetyl-CoA derivatives like carotenoids, fatty acids, and lipids. It also explores the functional differences between these isoenzymes, marking the first analysis of their relationship with carotenoid metabolism and mutual regulation.

## Materials and methods

### Yeast strains and culture conditions

*R. kratochvilovae* YM25235 was previously isolated from Chenghai Lake, Yunnan, China (Cui et al. 2016). Yeast strains were grown at 28 °C in yeast extract peptone dextrose (YPD: 1% (w/v) yeast extract, 2% (w/v) peptone, and 2% (w/v) glucose) media and synthetic complete (SC: 0.17% (w/v) yeast nitrogen base, 2% (w/v) glucose, 0.5% (w/v) ammonium sulfate, amino acid mixture, uracil added or removed as required) media. *Escherichia coli* strain DH5α was grown in Luria–Bertani (LB: 1% (w/v) NaCl, 1% (w/v) peptone, and 0.5% (w/v) yeast extract) media at 37 °C.

### sgRNA design and plasmid construction

Following previous research (Guo et al. 2022), deletion mutants were constructed in *R. kratochvilovae* (Ura3∆) via the CRISPR-Cas9 system. The guide RNA (gRNA) for genome editing was designed using the CRISPOR online platform (http://crispor.tefor.net/). The genomic sequences of *RkACS1* and *RkACS2* were retrieved from the *R. kratochvilovae* YM25235 genome (NCBI accession number: PRJNA739038). The 20-nucleotides gRNA was selected based on the high CRISPOR score and minimal off-target effects (Table S1). These gRNAs were synthesized by Sangon Biotech (Shanghai, China), and the sgRNA expression constructs were subsequently prepared.

To construct the complementary strains, we first obtained the cDNA of YM25235 using a previously described (He et al. 2023) and amplified the full-length coding sequences of *RkACS1* and *RkACS2* (Table S2, RkACS1-F1 and RkACS1-R1, RkACS2-F1 and RkACS2-R1). The PCR products were then subcloned into the pRG2034 vector (Guo et al. 2022; He et al. 2022) via the ClonExpress II One Step Cloning Kit (Vazyme, Nanjing, China) to generate the complementary plasmids pRGRkACS1 and pRGRkACS2.

### Yeast transformation and screening

The introduction of the sgRNA transcription plasmid into *R. kratochvilovae* (Ura3∆) was achieved through *Agrobacterium tumefaciens*-mediated transformation (Liu et al. 2013). The *RkACS1* and *RkACS2* deletion mutants were subsequently screened using SC-Ura media. The mutants were subsequently verified through PCR (Table S2, RkACS1-F1 and RkACS1-R1, RkACS2-F1 and RkACS2-R1) and sequencing. To generate the complementary strains, the plasmids pRGRkACS1 and pRGRkACS2 were introduced into the *RkACS1* and *RkACS2* deletion mutants, respectively, employing the same transformation method. Positive transformants were selected on YPD agar plates supplemented with G418 sulfate (30 μg/mL) and hygromycin B (150 μg/mL). To confirm the complementary strains, we designed the primers RkACS1-R2 and RkACS2-R2 at the fragment deletions of the *RkACS1* and *RkACS2* genes, respectively (Table S2), and their presence was confirmed via PCR (RkACS1-F1 and RkACS1-R2, RkACS2-F1 and RkACS2-R2) and sequencing.

### Assessing yeast growth on different carbon sources

For the spot assay, yeast strains were grown overnight in YPD liquid media at 28 °C until they reached the mid-log phase, after which they were washed with ddH_2_O, and the optical density at 600 nm (OD_600_) was adjusted to 1.0. The cells were serially diluted tenfold and spotted onto solid YPD and SC media containing 2% (w/v) glucose, sodium acetate, ethanol, or glycerol. The plates were then incubated at 28 °C for 2–5 days.

### Determination of the intracellular acetic acid

Yeast strains were cultured in 100 mL YPD liquid media at 28 °C, 160 rpm for 120 h. Cells were collected via centrifugation at 4 °C, 4500 rpm for 5 min, washed with PBS, snap-frozen in liquid nitrogen, and stored at –80 °C. The frozen samples were ground, and 0.2 g of each sample was mixed with 1 mL precooled ddH_2_O, shake well, sonicate in an ice for 15 min (300 W power, 5 s pulses, 10 s intervals), and centrifuged at 4 °C, 12000 rpm for 10 min. The supernatant was filtered (0.22 μm), and stored at 4 °C for analysis. Acetic acid content was determined using high-performance liquid chromatography (HPLC) (Rugthaworn et al. 2014) on an Agilent 1260 Infinity II and an Agilent Zorbax SB-C18 (250 mm × 4.6 mm, 5 μm) column. The mobile phases were A phase: 0.01 mol/L NaH_2_PO_4_ solution (pH = 2.98) and B phase: anhydrous methanol, with a ratio of A: B = 98:2. The detection wavelength was 210 nm, column temperature 25 °C, flow rate 1 mL/min, and injection volume 25 μL.

### Determination of intracellular acetyl-CoA levels

Yeasts were grown in 100 mL YPD liquid media at 28 °C, 160 rpm for 120 h, then centrifuged at 4 °C, 4500 rpm for 5 min, washed with PBS, aliquoted (0.1 g), and frozen in liquid nitrogen. Cell lysis and acetyl-CoA extraction were performed strictly following the manufacturer's protocol (Solarbio, Beijing, China). Quantification was achieved by measuring absorbance at 340 nm and calculating concentrations against a standard curve.

### Determination of total carotenoids

Yeast strains were cultured in 100 mL of YPD liquid media at 28 °C, 160 rpm for 120 h. then collected by centrifugation at 4 °C, 4500 rpm for 5 min, washed with PBS and freeze-dried. As described in the previous study (He et al. 2023), total carotenoids were extracted via acetone-methanol. Absorbance was measured at 450 nm, and concentrations were calculated to quantify carotenoid levels (Liaaen-Jensen and Jensen 1971).

### Determination of total lipids and fatty acids

Cells were collected and freeze-dried as described in this paper. As described in the previous study (He et al. 2023), total lipids were extracted using the Bligh–Dyer method (Bligh and Dyer 1959). Fatty acids were methyl esterified by the boron trifluoride-methanol and analyzed by gas chromatography–mass spectrometry (GC–MS) (Bhuiyan et al. 2014).

### Real-time quantitative polymerase chain reaction

Total RNA extraction and quantitative PCR (qPCR) analysis were performed as previously described (He et al. 2023). Primers, designed via the National Center for Biotechnology Information (NCBI) tool, are listed in Table S3. qPCR was conducted on a Bio-Rad CFX96 Fast Real-Time PCR System (Bio-Rad, Hercules, CA, USA) with ChamQ SYBR qPCR Master Mix (Vazyme, Nanjing, China). Small subunit rRNAs (*SSU rRNAs*) was used as the endogenous reference gene for the relative quantification of the expression of *RkACS1* and *RkACS2*, as well as genes related to carotenoid synthesis and fatty acid metabolism, using the 2^−ΔΔCT^ method.

### Western blot

Crude extracts were prepared as described in a previous study (He et al. 2019), and subjected to sodium dodecyl sulfate–polyacrylamide gel electrophoresis (SDS-PAGE) (Tanon, Shanghai, China), followed by transferred onto polyvinylidene difluoride (PVDF) membranes (Biosharp, Anhui, China). Immunoblotting was conducted in phosphate buffer solution (PBST) with 5% skim milk powder (BioFroxx, Einhausen, Germany). Primary antibodies used included histone H3 (Beyotime: AF0009), acetyl histone H3 (Lys9) (Beyotime: AG3948) and α-tubulin (ABclonal: AC012). The secondary antibody used was horseradish peroxidase-linked anti-mouse IgG (TransGen: HS201-01). Signals were detected via a Tanon 4600 luminescence image analyzer and quantified via enhanced chemiluminescence (ECL) (Tanon, Shanghai, China). The membranes were stripped and re-assayed according to the manufacturer's instructions (GenStar, Beijing, China). Grayscale values were analyzed via ImageJ software.

### Protein domain analysis

Protein domain analysis was performed using the CD-Search tool within the NCBI-hosted Conserved Domain Database (CDD). Default parameters were applied with an *E*-value threshold of 0.01, and functional annotations were prioritized through SMART, Pfam, and COG domain models. Results were displayed in a concise format.

### Statistical analysis

Data were analyzed via GraphPad Prism 9.5 software (GraphPad Software, CA, USA). Results are expressed as the means ± standard deviations (SDs), with at least triplicate experiments. Statistical comparisons were made using the Tukey test: * *p* < 0.05, ** *p* < 0.01, and *** *p* < 0.001.

## Results

### Deletion and restoration of the *RkACS1* and *RkACS2* genes in *R. kratochvilovae* YM25235

**Fig. 1:**
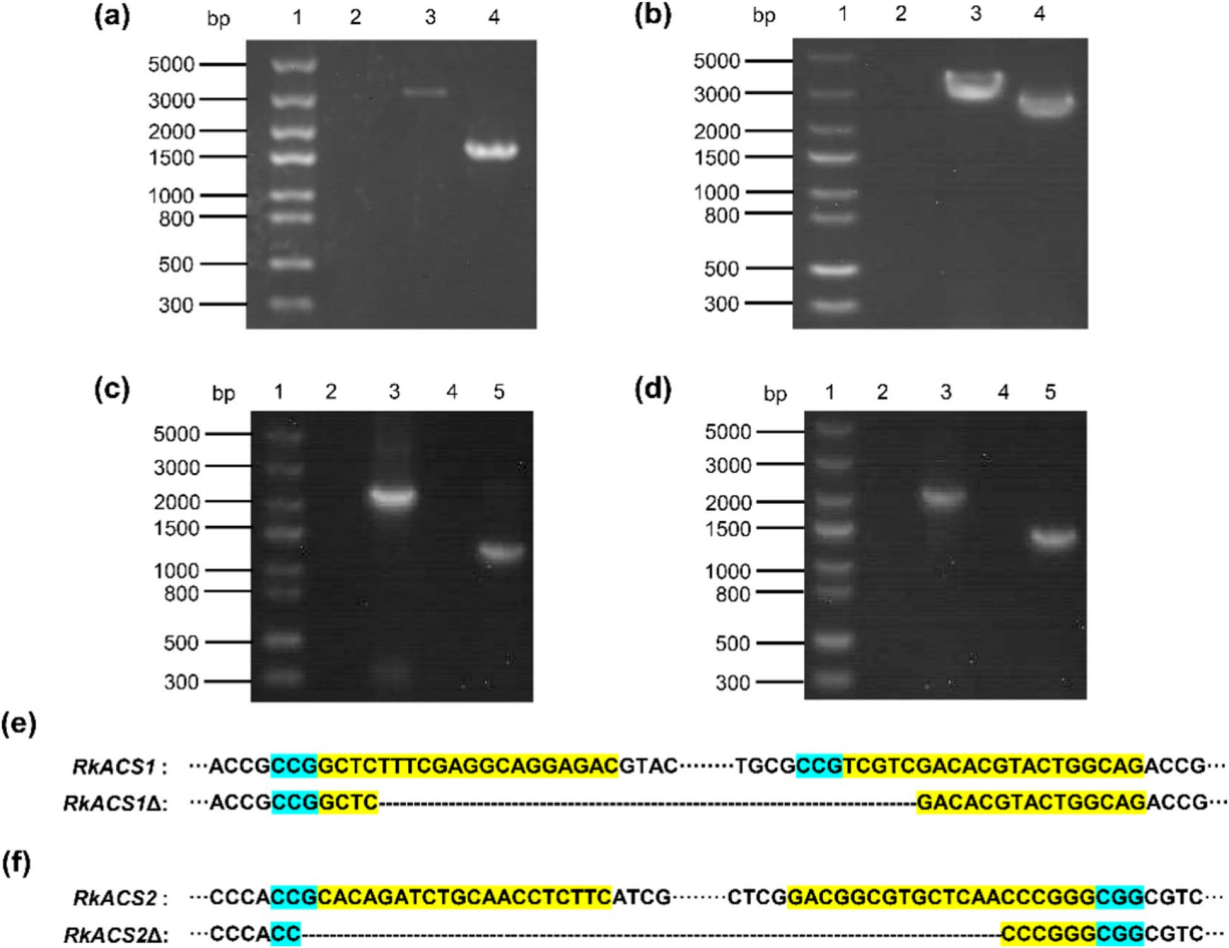
PCR and sequencing validation of deletion mutants and restoration strains. **a** Agarose gel analysis for *RkACS1* deletion mutant. 1. DNA marker; 2. Negative control; 3. PCR product of YM25235 amplified by the primers RkACS1-F1 and RkACS1-R1; 4. PCR product of YM25235RkACS1Δ was amplified with the primers RkACS1-F1 and RkACS1-R1. **b** Agarose gel analysis for *RkACS2* deletion mutant. 1. DNA marker; 2. Negative control; 3. PCR product of YM25235 was amplified with the primers RkACS2-F1 and RkACS2-R1; 4. PCR product of YM25235RkACS2Δ was amplified with the primers RkACS2-F1 and RkACS2-R1. **c** Agarose gel analysis of *RKACS1* complement strain. 1. DNA marker; 2. Negative control; 3. PCR product of YM25235 was amplified with the primers RkACS1-F1 and RkACS1-R2. 4. PCR product of YM25235RkACS1Δ was amplified with the primers RkACS1-F1 and RkACS1-R2. 5. PCR product of YM25235RkACS1Δ/pRGRkACS1 was amplified with the primers RkACS1-F1 and RkACS1-R2. **d** Agarose gel analysis of *RKACS2* complement strain. 1. DNA marker; 2. Negative control; 3. PCR product of YM25235 was amplified with the primers RkACS2-F1 and RkACS2-R2. 4. PCR product of YM25235RkACS2Δ was amplified with the primers RkACS2-F1 and RkACS2-R2. 5. PCR product of YM25235RkACS2Δ/pRGRkACS2 was amplified with the primers RkACS2-F1 and RkACS2-R2. **e** Sequencing of YM25235RkACS1Δ. **f** Sequencing of YM25235RkACS2Δ. gRNA sequence (yellow), PAM sequence (light blue), deletions (black dashes)

In this study, PCR amplification and sequencing confirmed that the *RkACS1* gene with a length of 3055 bp was targeted by Cas9 near the PAM site, resulting in the removal of a 1391 bp fragment from its genomic sequence and generating the *RkACS1* deletion strain YM25235RkACS1Δ (Fig. 1a, e). Similarly, the precise deletion of the 555 bp region in the *RkACS2* gene with a length of 3096 bp (Fig. 1b, f), successfully led to the generation of the *RkACS2* deletion strain YM25235RkACS2Δ. Additionally, PCR validation of complementary strains (Fig. 1c, d) demonstrated successful genes restoration: the target genes were detected in YM25235 and their respective complementary strains (YM25235RkACS1Δ/pRGRkACS1 and YM25235RkACS2Δ/pRGRkACS2), but absent in the deletion mutants.

### Effects of the *RkACS1* and *RkACS2* genes on the ability of YM25235 to utilize different carbon sources

**Fig. 2:**
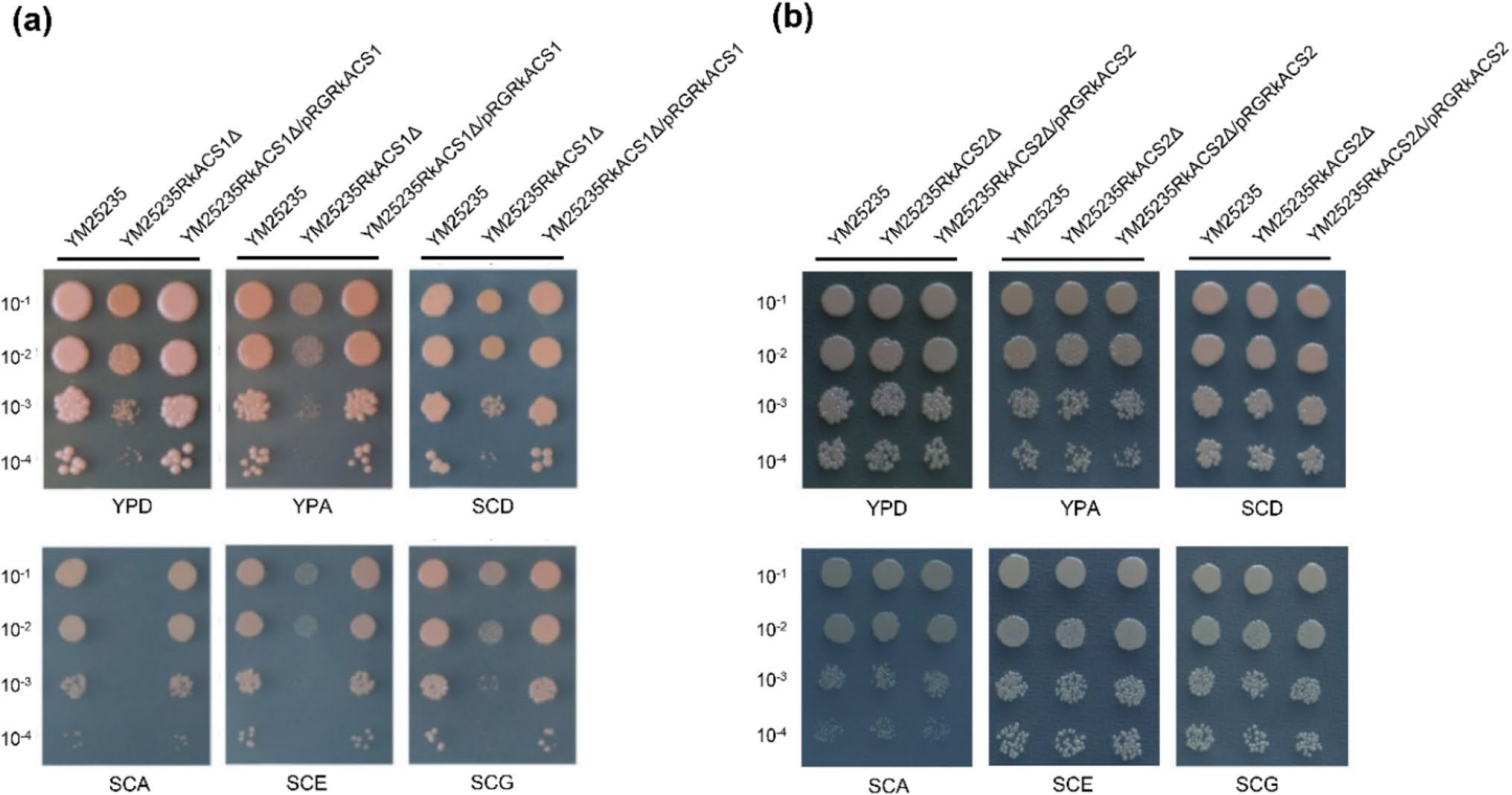
Growth of mutant strains on different carbon sources. **a** Growth of YM25235RkACS1Δ strain. **b** Growth of YM25235RkACS2Δ strain. Y: yeast extract; P: peptone; D: dextrose (glucose); A: sodium acetate; E: ethanol; G: glycerol; SC: synthetic complete medium

To investigate the effects of *RkACS1* and *RkACS2* deletion on carbon source utilization, the mutants were grown on solid media with different carbon sources. As shown in Fig. 2, compared to the wild-type YM25235, the growth of *RkACS1* gene deletion strain YM25235RkACS1Δ exhibited significantly restricted in glucose-based YPD medium, and its colonies were obviously smaller and thinner. When cultured in YPA medium containing sodium acetate as the sole carbon source, the growth impairment of the *RkACS1* deletion strain was further aggravated. The *RkACS1* deletion mutant failed to grow on SC media with acetate, grew poorly on ethanol and glycerol, and showed weaker growth on glucose. In contrast, the *RkACS2* mutant showed little growth difference on media with glucose, glycerol, ethanol, or acetate. These results indicate that *RkACS1* is essential for non-fermentable carbon sources utilization and cell growth. In sharp contrast, *RkACS2* showed no detectable impact on growth under all test carbon sources.

### Effects of the *RkACS1* and *RkACS2* genes on the intracellular acetic acid and acetyl-CoA levels in YM25235

Intracellular acetyl-CoA synthetase catalyzes the conversion of acetic acid to acetyl-CoA. We determined the intracellular acetic acid content by HPLC, and the results are shown in Table 1. The acetic acid content was greater in the deletion mutant strains YM25235RkACS1Δ (52.11 ± 1.80 mg/L) and YM25235RkACS2Δ (47.13 ± 1.50 mg/L) than in the wild-type strain YM25235 (38.70 ± 0.14 mg/L), with increases of 34.65% and 21.78%, respectively. However, after the restoration of RkACS1 or RkACS2, the expression levels in their complementary strains YM25235RkACS1Δ/pRGRkACS1 and YM25235RkACS2Δ/pRGRkACS2 were significantly increased compared with in the wild-type strain (Fig. S1), resulting in the intracellular acetic acid content decreased to 34.03 mg/L and 31.44 mg/L, which were 12.07% and 18.76% lower than the wild-type content, respectively. These findings suggest that deletion of both the *RkACS1* and *RkACS2* genes results in reduced cellular utilization of acetic acid, allowing acetic acid to accumulate in cells.

**Table 1:** Analysis of the intracellular acetic acid and acetyl-CoA content

Strain	Acetic acid content (mg/L)	Acetyl-CoA content (nmol/mg)
YM25235	38.70 ± 0.14	698.34 ± 15.45
YM25235RkACS1Δ	52.11 ± 1.80^***^	456.55 ± 12.09^***^
YM25235RkACS1Δ/pRGRkACS1	34.03 ± 1.09^&&&^	727.60 ± 15.84^&&&^
YM25235RkACS2Δ	47.13 ± 1.50^***^	521.13 ± 10.44^***^
YM25235RkACS2Δ/pRGRkACS2	31.44 ± 1.11^**###^	898.33 ± 8.64^***###^

Symbols indicate comparisons: “*”, compared with YM25235; “&”, compared with YM25235RkACS1Δ; “#”, compared with YM25235RkACS2Δ

Next, we also measured the intracellular acetyl-CoA levels, as shown in Table 1, deletion of both *RkACS1* and *RkACS2* resulted in a significant decrease in acetyl-CoA levels in YM25235. Compared with those in the wild-type strain YM25235 (698.34 ± 15.45 nmol/mg), the levels were 34.62% lower in the deletion mutant YM25235RkACS1Δ (456.55 ± 12.09 nmol/mg) and 25.37% lower in YM25235RkACS2Δ (521.13 ± 10.44 nmol/mg). Notably, the expression of *RkACS1* or *RkACS2* in their complementary strains higher than in wild-type strain, thereby enhancing the conversion of acetic acid to acetyl-CoA (Fig. S1).

### Effects of the *RkACS1* and *RkACS2* genes on carotenoid synthesis in YM25235

Acetyl-CoA is a precursor for the biosynthesis of carotenoids. To investigate the impact of *RkACS1* and *RkACS2* genes deletion on carotenoid biosynthesis in YM25235, the carotenoid contents were measured. The results (Table 2) revealed that the carotenoid contents in the YM25235RkACS1Δ mutant (3.55 ± 0.12 mg/g DCW) was significantly lower than that in the wild-type strain YM25235 (4.45 ± 0.13 mg/g DCW), a decrease of 20.22%. In contrast, the YM25235RkACS2Δ (4.24 ± 0.20 mg/g DCW) mutant did not show a significant difference from the wild-type strain. However, complementation of the *RkACS1* and *RkACS2* genes (7.28 ± 0.11 mg/g DCW and 6.25 ± 0.18 mg/g DCW) restored carotenoid biosynthesis to levels higher than the wild-type, with increases of 63.59% and 40.45%, respectively.

**Table 2:** Analysis of carotenoid content

Strain	Carotenoid content (mg/g DCW)
YM25235	4.45 ± 0.13
YM25235RkACS1Δ	3.55 ± 0.12^***^
YM25235RkACS1Δ/pRGRkACS1	7.28 ± 0.11^***&&&^
YM25235RkACS2Δ	4.24 ± 0.20
YM25235RkACS2Δ/pRGRkACS2	6.25 ± 0.18^***###^

DCW: dry cell weight; Symbols indicate comparisons: “*”, compared with YM25235; “&”, compared with YM25235RkACS1Δ; “#”, compared with YM25235RkACS2Δ

**Fig. 3:**
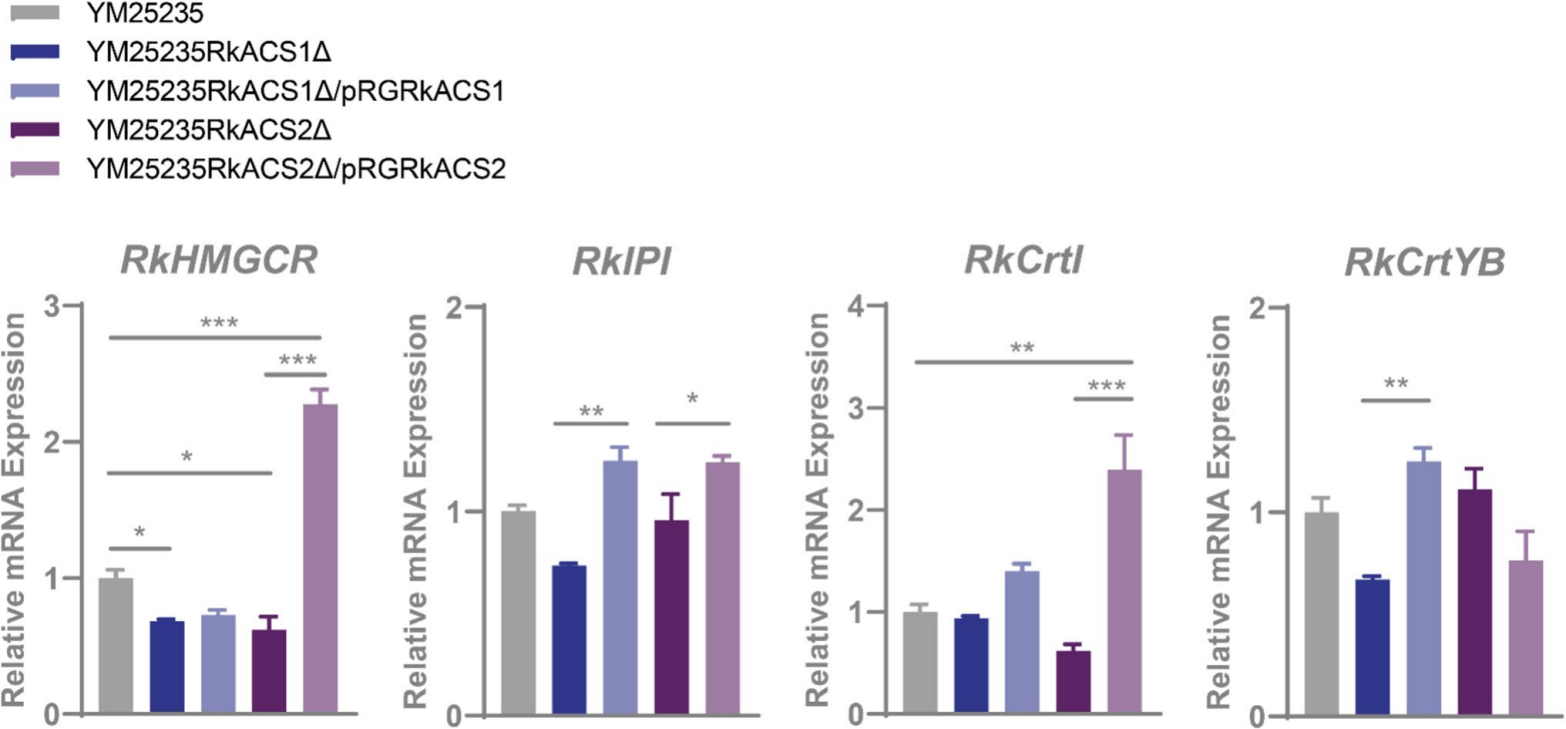
Effects of the *RkACS1* and *RkACS2* genes on the transcript levels of carotenoid synthesis-related genes. The relative quantification of carotenoid biosynthesis related genes expression was performed using the 2^−ΔΔCT^ method, with small subunit rRNAs (*SSU rRNAs*) as endogenous reference control. *RkHMGCR*: HMG-CoA reductase gene; *RkIPI*: isopentenyl diphosphate isomerase gene; *RkCrtI*: phytoene dehydrogenase gene; *RkCrtYB*: phytoene synthase/lycopene cyclase gene

In addition, the qPCR results confirmed (Fig. 3) that the expression of the *RkHMGCR*, *RkIPI*, and *RkCrtYB* genes, which encode enzymes critical for the MVA pathway, the isoprenoid synthesis pathway, and the carotenoid biosynthesis pathway, respectively, was significantly suppressed in the deletion mutant YM25235RkACS1Δ. The expression of the *RkHMGCR* and *RkCrtI* genes was inhibited in the deletion mutant YM25235RkACS2Δ. However, after the complementation of the *RkACS1* and *RkACS2* genes, the expression of most genes involved in carotenoid synthesis were restored and even higher than that in the wild-type strain. *RkIPI*, *RkCrtI* and *RkCrtYB* were up-regulated in the *RkACS1* complementary strain, while *RkHMGCR*, *RkIPI* and *RkCrtI* were up-regulated in the *RkACS2* complementary strain. These findings indicate that *RkACS1* and *RkACS2* can regulate the expression of genes related to carotenoid biosynthesis in YM25235.

### Effects of the *RkACS1* and *RkACS2* genes on lipid and fatty acid metabolism in YM25235

Lipids and fatty acids are also important acetyl-CoA-derived compounds. We examined the levels of lipids and fatty acids in the cells. The results (Table 3) revealed that lipid accumulation in the deletion mutant YM25235RkACS1Δ (14.73 ± 0.28% DCW) significantly increased by 140.68% compared with that in the wild-type strain YM25235 (6.12 ± 0.66% DCW), whereas it decreased to 51.80% in the complementary strain YM25235RkACS1Δ/pRGRkACS1 (9.29 ± 0.15% DCW). In contrast, the lipid content of the deletion mutant YM25235RkACS2Δ (5.09 ± 0.09% DCW) was 16.83% lower than that of the wild-type, the complementary strain YM25235RkACS2Δ/pRGRkACS2 (9.16 ± 0.29% DCW) increased to 49.67%. Fatty acid content measurements (Table 4) revealed that *RkACS1* knockout in YM25235 increased the total fatty acid content by 114.13%, with significant increases in almost all components, including palmitic acid (C16:0), palmitoleic acid (C16:1), oleic acid (C18:1), linoleic acid (LA, C18:2), and α-linolenic acid (ALA, C18. 3). *RkACS2* knockout reduced the synthesis of total fatty acids by 13.58%, which was associated mainly with palmitic acid (C16:0) and oleic acid (C18:1).

**Table 3:** Analysis of total lipids

Strain	Lipid accumulation (% DCW)
YM25235	6.12 ± 0.66
YM25235RkACS1Δ	14.73 ± 0.28^***^
YM25235RkACS1Δ/pRGRkACS1	9.29 ± 0.15 ^***&&&^
YM25235RkACS2Δ	5.09 ± 0.09
YM25235RkACS2Δ/pRGRkACS2	9.16 ± 0.29^***###^

DCW: dry cell weight; Symbols indicate comparisons: “*”, compared with YM25235; “&”, compared with YM25235RkACS1Δ; “#”, compared with YM25235RkACS2Δ

**Table 4:** Analysis of fatty acid composition

Strain	Total fatty acids (mg/g)	Fatty acid composition (mg/g)					
		C16:0	C16:1	C18:0	C18:1OA	C18:2LA	C18:3 ALA
YM25235	32.63 ± 0.70	3.77 ± 0.13	0.89 ± 0.35	0.19 ± 0.01	18.94 ± 0.46	7.82 ± 0.06	1.00 ± 0.02
YM25235RkACS1Δ	69.87 ± 0.05^***^	15.11 ± 0.01^***^	1.63 ± 0.05^***^	0.12 ± 0.01^**^	39.44 ± 0.39^***^	10.01 ± 0.39^***^	2.44 ± 0.02^***^
YM25235RkACS1Δ/pRGRkACS1	45.60 ± 0.46^***&&&^	7.74 ± 0.14^***&&&^	1.44 ± 0.14^**^	0.92 ± 0.03^***&&&^	25.24 ± 0.22^***&&&^	8.84 ± 0.04^***&&&^	1.43 ± 0.02^***&&&^
YM25235RkACS2Δ	28.20 ± 1.58	3.28 ± 0.27	0.70 ± 0.04	0.19 ± 0.02	14.76 ± 0.7^**^	7.89 ± 0.49	1.19 ± 0.06^*^
YM25235RkACS2Δ/pRGRkACS2	48.02 ± 1.25^***###^	5.79 ± 0.25^***###^	1.38 ± 0.05^###^	0.25 ± 0.00^*#^	26.81 ± 1.24^***###^	11.43 ± 0.13^***###^	2.54 ± 0.09^***###^

Symbols indicate comparisons: “*”, compared with YM25235; “&”, compared with YM25235RkACS1Δ; “#”, compared with YM25235RkACS2Δ

**Fig. 4:**
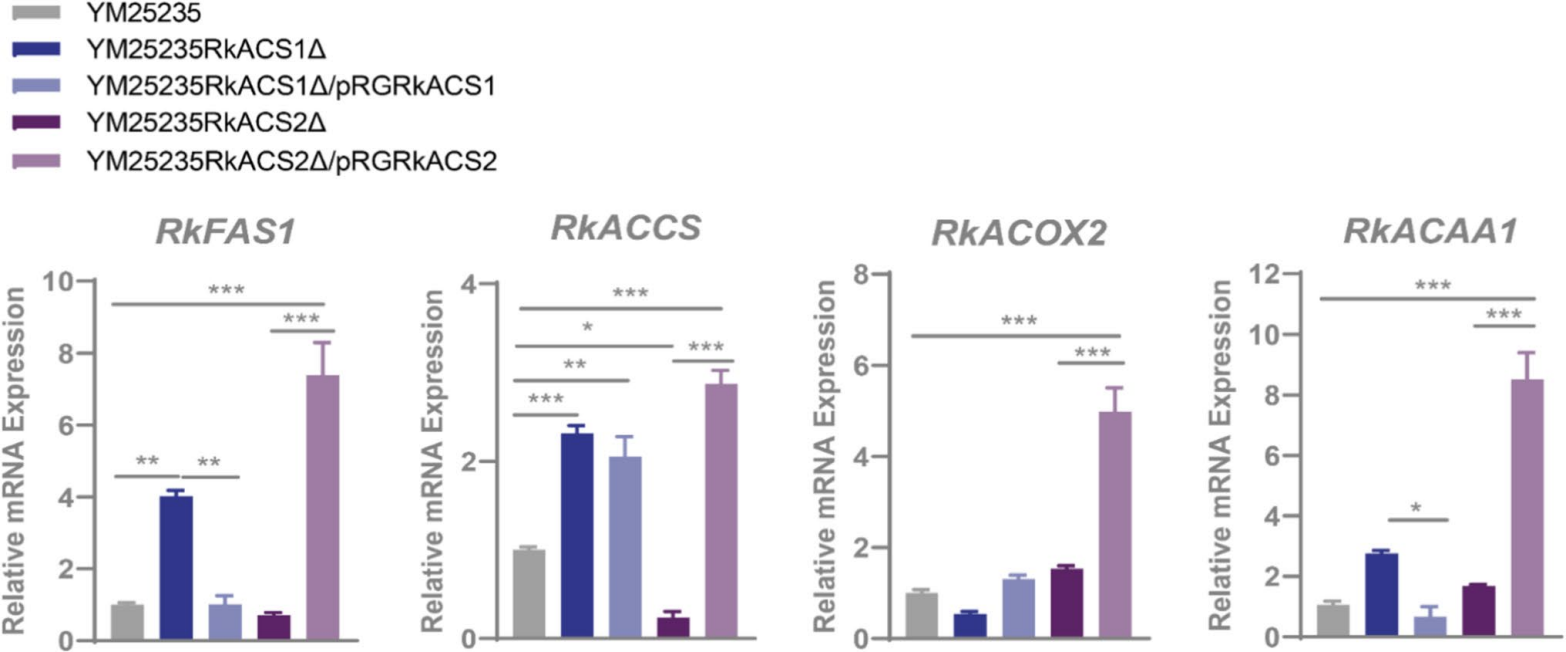
Effects of the *RkACS1* and *RkACS2* genes on the transcript levels of genes related to fatty acid metabolism. The relative quantification of fatty acid metabolism related genes expression was performed using the 2^−ΔΔCT^ method, with small subunit rRNAs (*SSU rRNAs*) as endogenous reference control. *RkFAS1*: fatty acid synthase gene; *RkACCS*: acetyl-CoA carboxylase gene; *RkACOX2*: fatty acyl-CoA oxidase gene; *RkACAA1*: 3-oxoacyl-CoA thiolase gene

The results of the transcript level measurements (Fig. 4) also confirmed that the expression of the *RkFAS1* and *RkACCS* genes involved in fatty acid synthesis was up-regulated in the deletion mutant YM25235RkACS1Δ, whereas the expression of the *RkACOX2* genes involved in fatty acid catabolism was down-regulated. The opposite was true for the deletion mutant YM25235RkACS2Δ, in which the expression of the *RkFAS1* and *RkACCS* genes related to fatty acid synthesis was down-regulated, whereas the expression of *RkACOX2* and *RkACAA1*, genes related to fatty acid degradation, was up-regulated. These results indicate that both *RkACS1* and *RkACS2* can affect fatty acid metabolism in YM25235, but the two genes are functionally distinct.

### Interconnection between *RkACS1* and *RkACS2* in YM25235

**Fig. 5:**
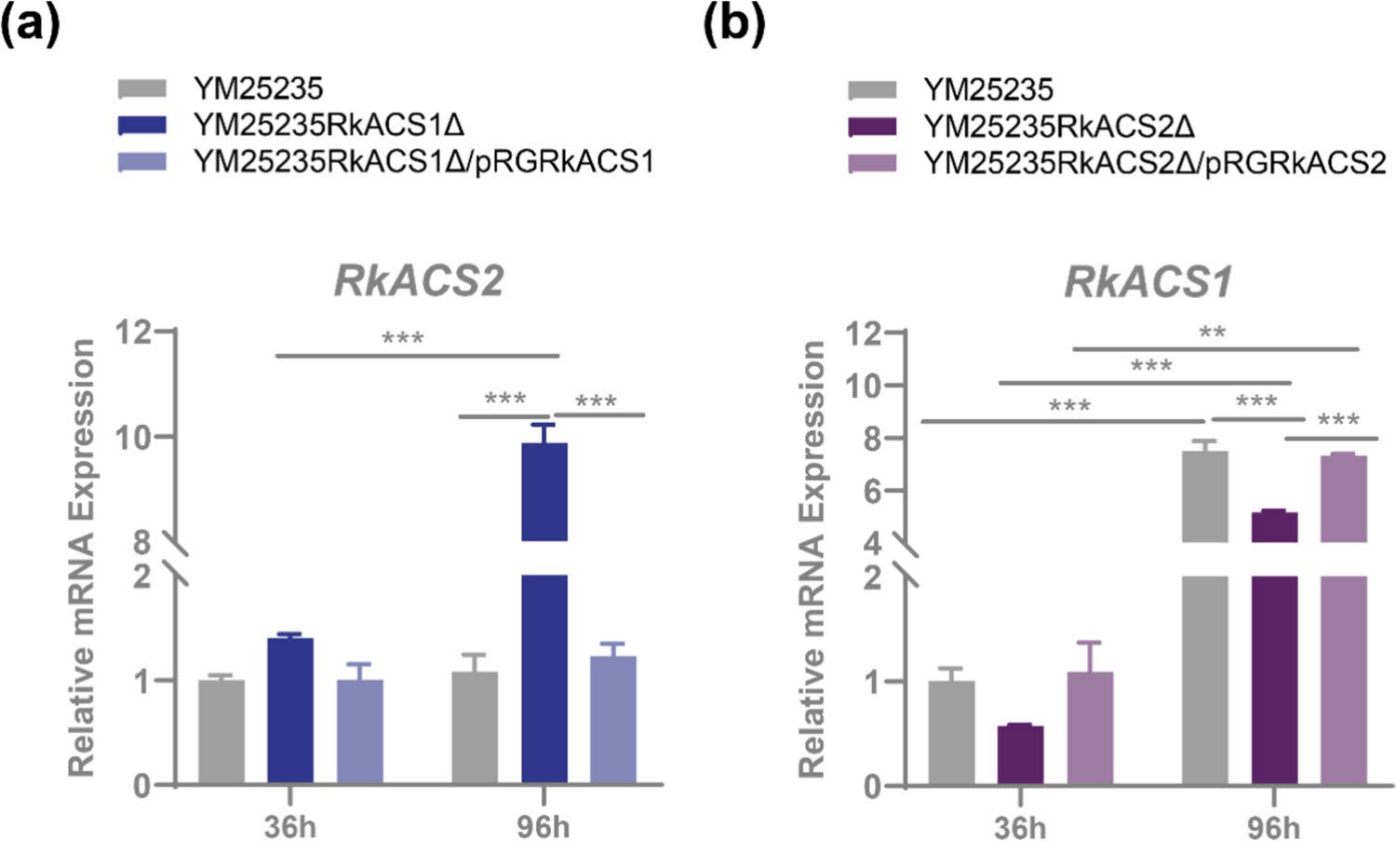
Interconnection of the *RkACS1* and *RkACS2* genes. The relative quantification of the expression of *RkACS1* and *RkACS2* was performed using the 2^−ΔΔCT^ method, with small subunit rRNAs (*SSU rRNAs*) as endogenous reference controls. **a** Expression of the *RkACS2* gene in the deletion mutant YM25235RkACS1Δ. **b** Expression of the *RkACS1* gene in the deletion mutant YM25235RkACS2Δ

To elucidate the interconnection between RkACS1 and RkACS2, we analyzed the transcript levels of *RkACS1* and *RkACS2* under glucose starvation (96 h) and non-starvation (36 h) conditions on the basis of a previous study (He et al. 2023). Transcript level analysis showed that *RkACS1* in the wild-type strain YM25235 exhibited glucose-dependent regulation, with expression being up-regulated 7.51-fold under glucose starvation but showing repression under the presence of glucose (Fig. 5b), whereas *RkACS2* expression remained stable and unaffected by glucose availability (Fig. 5a). Compared with that in the wild-type, the *RkACS2* gene was highly expressed in the deletion mutant YM25235RkACS1Δ, especially under glucose starvation condition, at levels up to 9.89-fold (Fig. 5a), whereas *RkACS1* gene expression was down-regulated in the deletion mutant YM25235RkACS2Δ (Fig. 5b). The results suggest that in YM25235, the expression of the *RkACS1* gene is regulated by glucose, while the *RkACS2* gene remains stably expressed and is not influenced by glucose levels. Additionally, the deletion of *RkACS1* led to increased expression of the isozyme gene *RkACS2* under glucose starvation condition, whereas the deletion of *RkACS2* resulted in reduced *RkACS1* expression.

### RkACS2 is involved in the regulation of histone acetylation in YM25235

**Fig. 6:**
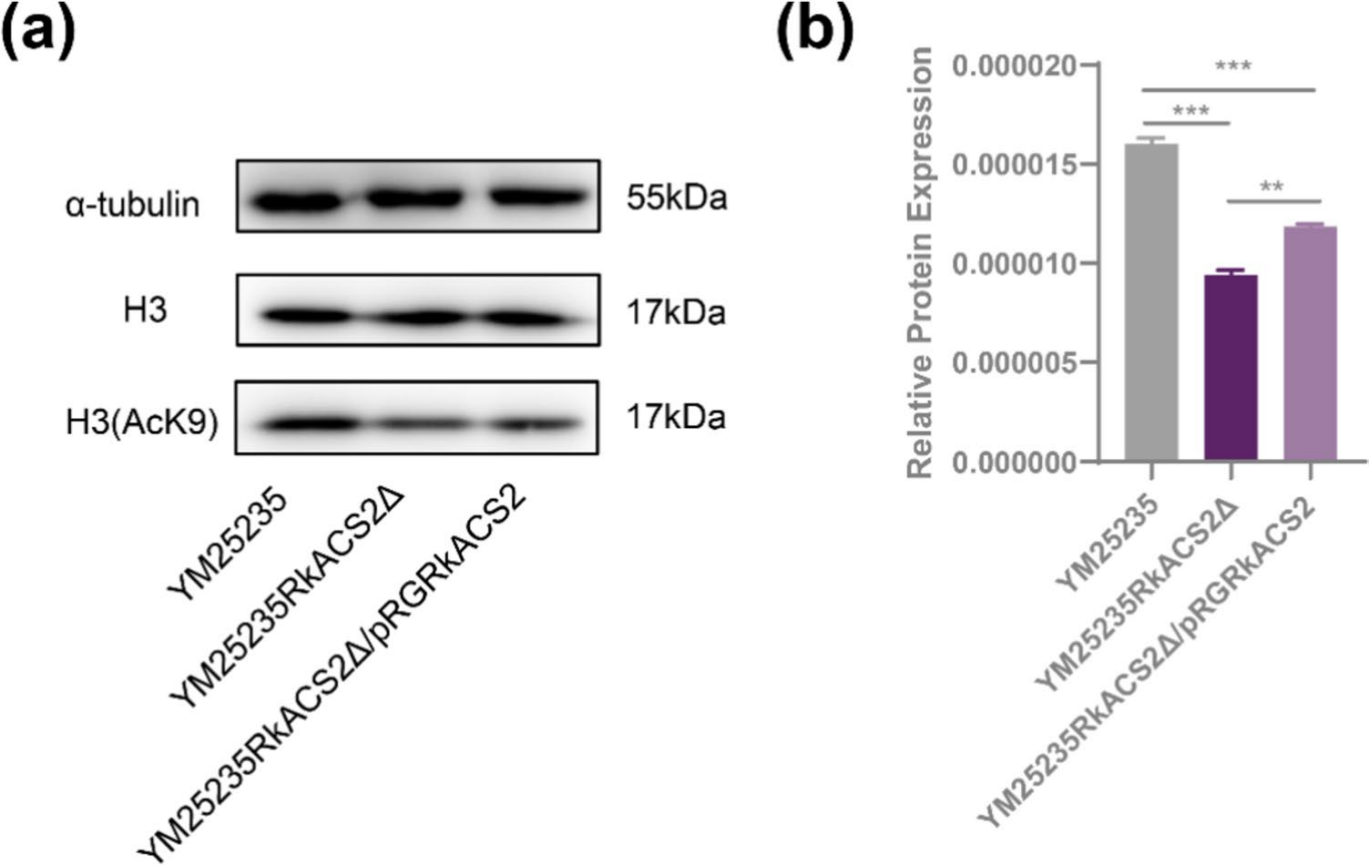
Levels of acetylation of histone H3. **a** Immunoblotting of crude extracts of *RkACS2* mutant. **b** Relative expression of the H3 (ACK9) protein. The grayscale in (a) was calculated via ImageJ, and the relative expression of H3 (AcK9) was calculated from H3 (AcK9)/H3/α-tubulin

Acetyl-CoA produced by ACS2 is critical for histone acetylation (Takahashi et al. 2006). To gain a clearer understanding of the impact of *RkACS2* gene deletion on histone acetylation levels in YM25235, immunoblotting was conducted using an anti-acetyl-histone H3 (Lys9) (H3-AcK9) antibody. As depicted in Fig. 6, the deletion of the *RkACS2* gene resulted in a 41.19% decrease in histone acetylation levels. Following the reintroduction of *RkACS2*, the relative level of H3-AcK9 increased but remained below that of the YM25235 strain. These findings indicate that the acetyl-CoA produced by RkACS2 is one of the important sources of acetyl group donors for histone acetylation in *R. kratochvilovae* YM25235.

## Discussion

Acetyl-CoA is a vital precursor for the biosynthesis of natural products like carotenoids, fatty acids, and lipids (Zhang et al. 2021). In *S. cerevisiae*, the ACS1 and ACS2 isoenzymes are key contributors to acetyl-CoA biosynthesis, and the dual deletion of *ACS1* and *ACS2* leads to cells unable to survive (van den Berg et al. 1996). However, there are notable distinctions between these two isoenzymes: *ACS1* encodes an aerobic form of the enzyme, whereas *ACS2* encodes a non-aerobic form (van den Berg et al. 1996), which exhibit disparate preferences for carbon sources. ACS1 is associated with growth on non-fermentable carbon sources such as acetate or ethanol (Liu et al. 2016; Van den Berg and Steensma 1995), which has also been confirmed in *R. kratochvilovae* YM25235 (Fig. 2a). The *RkACS1* gene deletion mutant exhibits growth defects on acetate and significantly attenuated growth on ethanol, glycerol, and glucose. Similar to ACS1 and ACS2 in *S. cerevisiae*, RkACS1 has an acetate-binding site, and it exhibits a high affinity for acetate, whereas RkACS2 lacks this binding site (Table S4). This indicates that the acetyl-CoA synthetase encoded by *ACS1* is functionally conserved across different yeast species and is crucial for the efficient utilization of non-fermentable carbon sources, including acetate, ethanol, and glycerol. In contrast to *S. cerevisiae* and *Rhodosporidium diobovatum* where ACS2 serves as a key enzyme for glucose utilization (Liu et al. 2016; Van den Berg and Steensma 1995), the deletion of *RkACS2* in *R. kratochvilovae* YM25235 did not impair growth on any tested carbon sources (Fig. 2b). This phenotypic divergence may arise from species-specific regulatory divergence between *R. kratochvilovae* and *R. diobovatum* clades, and potential functional redundancy of RkACS1, though specific mechanisms require further investigation. These results suggest that RKACS1 plays a more conserved role in the utilization of non-fermentative carbon sources, whereas RKACS2 is not specific for carbon sources in *R. kratochvilovae* YM25235 compared to its homologues in *S. cerevisiae* and *R. diobovatum*.

The ACS increases the supply of acetyl-CoA in cells by catalyzing the conversion of acetate to acetyl-CoA (Liu et al. 2016). In this study, we observed that deletion of either the *RkACS1* or *RkACS2* gene in *R. kratochvilovae* YM25235 led to an accumulation of intracellular acetic acid and a reduction in acetyl-CoA levels (Table 1). This is in line with our previous findings showing that *ACS1* and *ACS2* enhance acetyl-CoA levels under glucose starvation (He et al. 2023). However, RkACS1 contributes significantly more to the cellular acetyl-CoA pool than RkACS2. This is attributed to the more conserved function of RkACS1 and its higher affinity for the substrate acetate, allowing it to convert acetate (a non-fermentable carbon source) to acetyl-CoA more effectively.

Carotenoids, as high-value acetyl-CoA derivatives, are significant metabolites of red yeasts (Zhang et al. 2022, 2021). Changes in the intracellular acetyl-CoA pool directly affect the production of downstream carotenoids. In this study, we found that deletion of either the *RkACS1* or *RkACS2* gene in *R. kratochvilovae* YM25235 caused a decrease in carotenoid biosynthesis (Table 2), alongside reduced transcription levels of key genes in the MVA pathway, isoprenoid synthesis pathway, and carotenoid biosynthesis pathway (Fig. 3). However, *RkACS1* exhibited a more pronounced impact on carotenoid biosynthesis (Table 2). When glucose becomes scarce, the inhibitory effect of glucose on RkACS1 was removed, and the dependence of cells on RkACS1 is significantly higher than that on RkACS2 (Fig. 5). At this time, cells will change their metabolic strategies and start to utilize non-fermentable carbon sources, including acetate, to maintain basic metabolic needs. The high expression of *RkACS1* provides a more supply of acetyl-CoA for carotenoid synthesis, and carotenoids help to alleviate the oxidative damage caused by glucose starvation. Given that carotenoids are high-value compounds (Widomska and Subczynski 2019), regulating the expression of *RkACS1* to increase the yield of carotenoids may provide novel strategies for biosynthesis on an industrial scale.

Lipids are also essential derivatives of acetyl-CoA (Zhang et al. 2021). Acetyl-CoA is converted to fatty acids and complex lipids via a series of reactions in the cytoplasm (Zhang et al. 2022). In this study, it was observed that deletion of the *RkACS1* gene in *R. kratochvilovae* YM25235 led to an increased accumulation of both fatty acids and lipids (Tables 3 and 4), while also promoting the transcription of genes involved in fatty acid synthesis (Fig. 4). This may be attributed to the up-regulation of the isoenzyme RkACS2 in response to the loss of RkACS1 (Fig. 5a), with RkACS2 influencing the expression of genes related to fatty acid biosynthesis (Hiesinger et al. 1997), thereby directing carbon flux from acetyl-CoA toward lipid synthesis. Consistent with our hypothesis, deletion of *RkACS2* in *R. kratochvilovae* YM25235 resulted in decreased fatty acid and lipid accumulation (Tables 3 and 4), while inducing the transcription of genes related to fatty acid β-oxidation (Fig. 4). These findings further indicates that RkACS2, rather than RkACS1, primarily catalyzes the production of acetyl-CoA involved in fatty acid and lipid biosynthesis. Additionally, both ACS1 and ACS2 are capable of activating free fatty acids into acyl-CoA esters, providing precursors for either lipid synthesis or fatty acid β-oxidation (Black and DiRusso 2007; Tenagy et al. 2015). In *R. kratochvilovae* YM25235, while both isoenzymes possessed acyl-activating enzyme (AAE) activity (Table S4), only the deletion of *RkACS1* resulted in fatty acid accumulation (Table 4). This is likely due to the differing substrate preferences of ACS1 and ACS2: ACS1 favors long-chain fatty acids (C12-C20), while ACS2 prefers medium- and short-chain fatty acids (Black and DiRusso 2007; Tenagy et al. 2015). As long-chain fatty acids predominate in *R. kratochvilovae* YM25235 (Table 4), deletion of *RkACS1* hinders the activation of fatty acids for β-oxidation. In summary, RkACS1 and RkACS2 play distinct roles in the lipid metabolism of *R. kratochvilovae* YM25235, with RkACS1 primarily involved in degradation processes, while RkACS2 is closely associated with biosynthesis. From an industrial perspective, enhancing *RkACS2* expression while suppressing *RkACS1* could be an effective strategy to boost lipid production in oleaginous microorganisms. It is noteworthy that excessive RkACS2 activity may disrupt lipid homeostasis, potentially leading to the accumulation of lipid peroxides (such as malondialdehyde (MDA)) (Black and DiRusso 2007). Therefore, in the process of biological production, precisely regulating its enzymatic activity is crucial for reducing the risk of metabolic imbalance.

The ACS2 enzyme is also localized in the nucleus, where it activates acetate into acetyl-CoA, providing the necessary precursor for histone acetylation (Takahashi et al. 2006). Acetylation of lysine residues is a common post-translational modification of histones (including histones H2 A, H2B, H3 and H4) (Trefely et al. 2020). Histone acetylation is crucial for cell cycle regulation, DNA repair, gene expression, and gene silencing (He et al. 2019). In *R. kratochvilovae* YM25235, deletion of the *RkACS2* gene resulted in reduced acetylation of lysine residues on histone H3 (Fig. 6), demonstrating that nuclear acetyl-CoA synthesized by RkACS2 is essential for histone acetylation. Additionally, the deletion of *RkACS2* led to the down-regulation of the isoenzyme RkACS1 and genes involved in carotenoid and lipid synthesis (Figs. 3 and 4), suggesting that RkACS2 is a crucial enzyme in nuclear metabolism, affecting both histone acetylation and gene expression.

Notably, there is also a reciprocal regulatory relationship between RkACS1 and RkACS2. In wild-type *R. kratochvilovae* YM25235, *RkACS1* expression is suppressed in the presence of glucose (Fig. 5b), whereas *RkACS2* maintains stable expression regardless of glucose availability (Fig. 5a), consistent with previous studies (Liu et al. 2016; Van den Berg and Steensma 1995). Deletion of *RkACS1* significantly induced the up-regulation of *RkACS2* expression, particularly under glucose starvation (Fig. 5a). This phenomenon may be attributed to functional redundancy between the two isozymes, wherein the absence of *RkACS1* enables *RkACS2* up-regulation to compensate for acetyl-CoA biosynthesis. Conversely, in the *RkACS2* deletion strain, the expression of *RkACS1* was decreased (Fig. 5b). This might be because the deletion of *RkACS2* led to a reduction in the supply of acetyl groups in the nucleus, thereby affecting the expression of *RkACS1*. Despite these findings, many aspects of the functional differences and interactions between RkACS1 and RkACS2 remain unclear. Further experimental research is needed to gain a more complete understanding of their roles in metabolic regulation.

Overall, this study clearly demonstrates that RkACS1 and RkACS2 exhibit different physiological functions in carbon source utilization, carotenoid biosynthesis, lipid metabolism, and acetylation regulation in *R. kratochvilovae* YM25235. (1) Under glucose starvation, RkACS1 can promote the utilization of acetic acid and fatty acid β-oxidation to provide acetyl-CoA, which in turn provides an indispensable precursor for other metabolic processes, such as the biosynthesis of carotenoids; (2) The acetyl-CoA produced by RkACS2 is mainly involved in acetylation to regulate the expression of relevant genes, such as genes related to fatty acid synthesis; (3) Functional redundancy and mutual regulation exist between the RkACS1 and RkACS2 isozymes. From an industrial production perspective, carrying out corresponding genetic engineering modifications targeting the functional specificity of RkACS1 and RkACS2 is a good strategy to effectively improve the production efficiency of acetyl-CoA derivatives.

## Supplementary Information

Below is the link to the electronic supplementary material.

Supplementary file1 (PDF 174 KB)

Supplementary file2 (PDF 82 KB)

Supplementary file3 (XLSX 12 KB)

## Data Availability

No datasets were generated or analysed during the current study.
