# Supplementary material for: Divergent roles of the acetyl-CoA synthetases RkACS1 and RkACS2 in carotenoid and lipid biosynthesis in Rhodosporidium kratochvilovae
Source: Appl Microbiol Biotechnol. 2025 Jun 7;109:140. doi: 10.1007/s00253-025-13534-x (PMC12145312; doi:10.1007/s00253-025-13534-x)
Supplement: Supplementary file 1 [file 253_2025_13534_MOESM1_ESM.pdf]

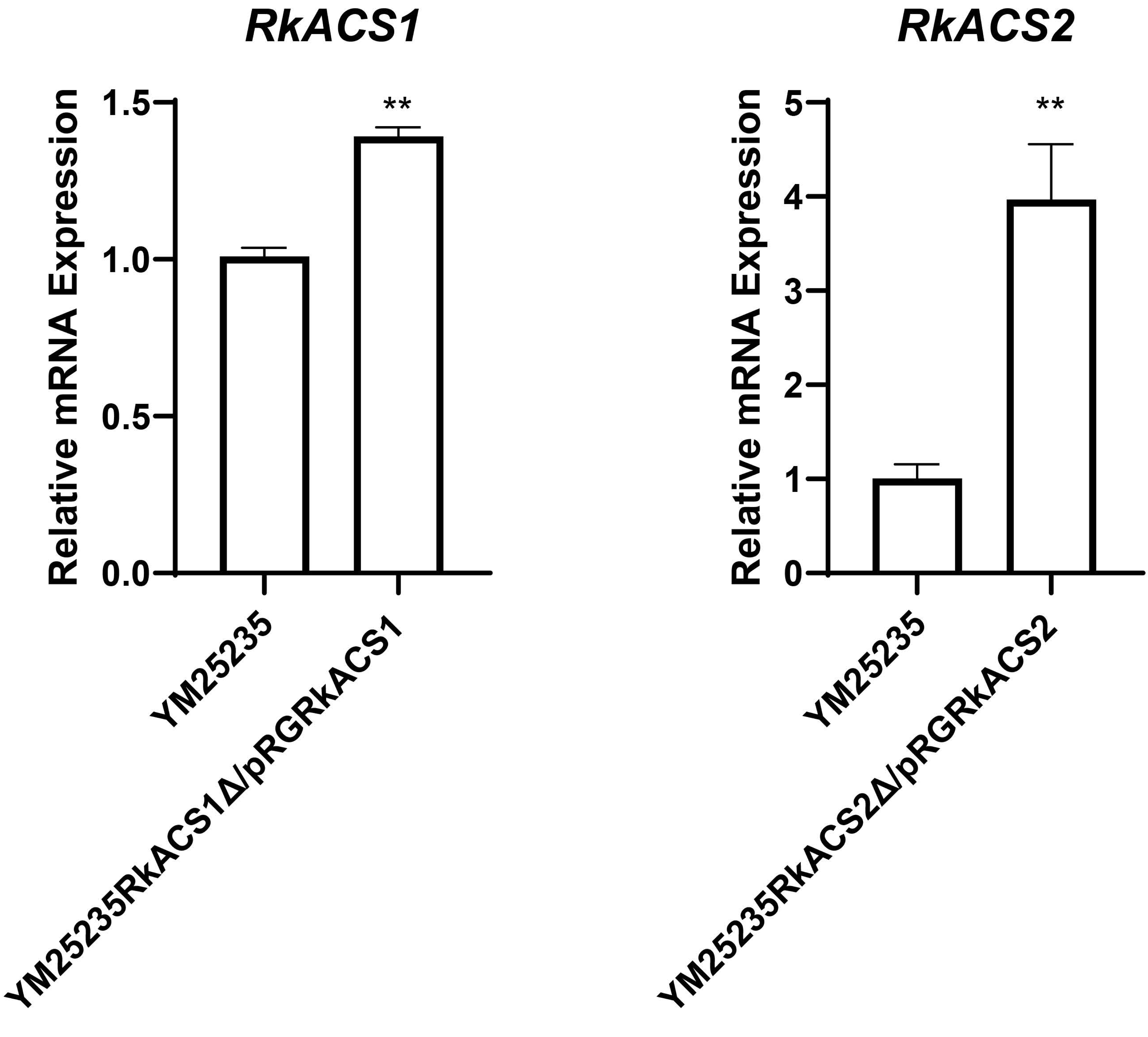
RkACS1
RkACS2
Relative mRNA Expression
1.5
1.0
0.5
0.0
5
4
3
2
1
0
**
**
YM25235
YM25235RkACS1Δ/pRGRkACS1
YM25235RkACS2Δ/pRGRkACS2
