# Supplementary figures and images for: Divergent roles of the acetyl-CoA synthetases RkACS1 and RkACS2 in carotenoid and lipid biosynthesis in Rhodosporidium kratochvilovae
Source: Appl Microbiol Biotechnol. 2025 Jun 7;109(1):140. doi: 10.1007/s00253-025-13534-x (PMC12145312; doi:10.1007/s00253-025-13534-x)

## *RkACS1*

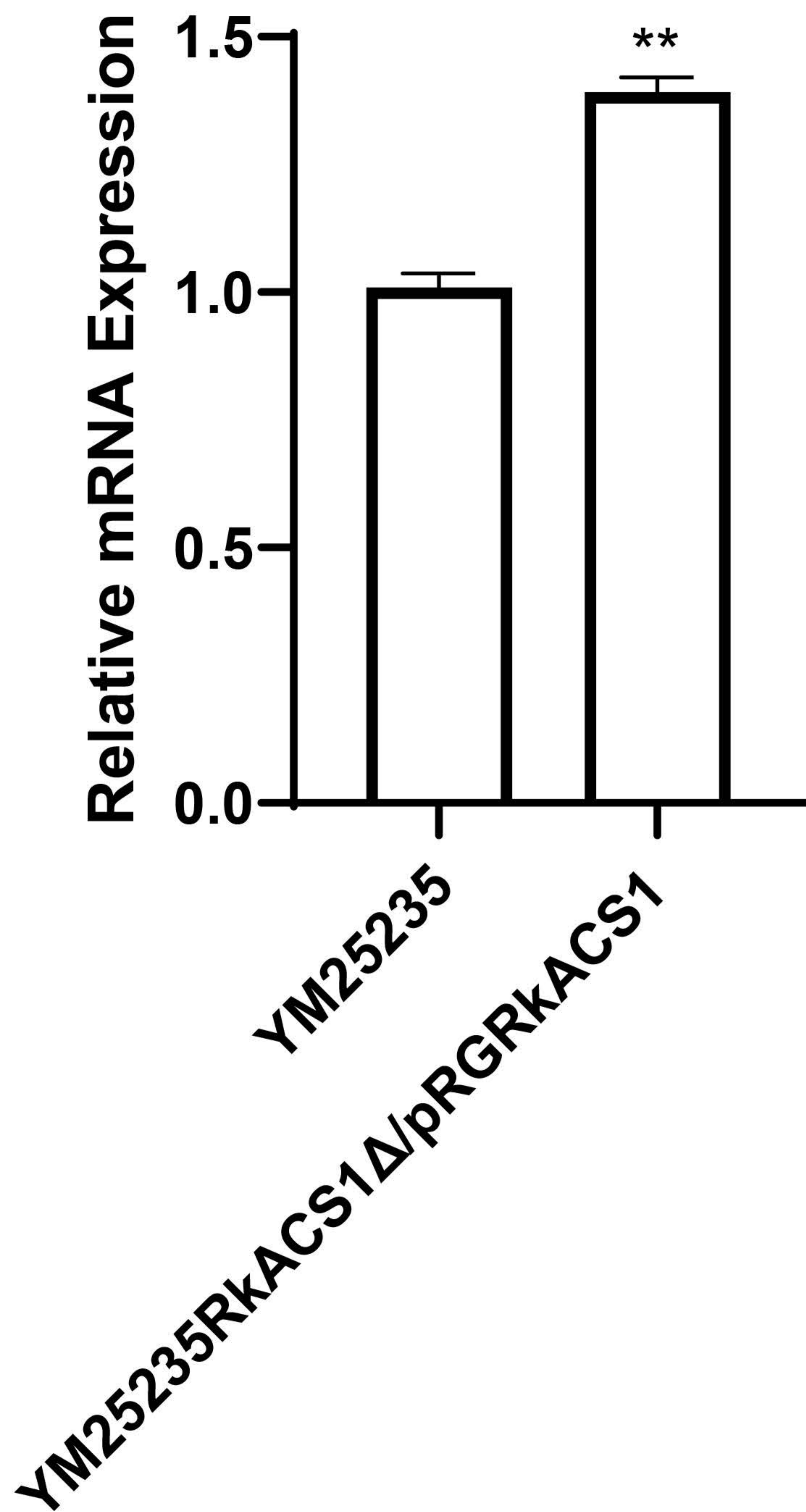

## *RkACS2*

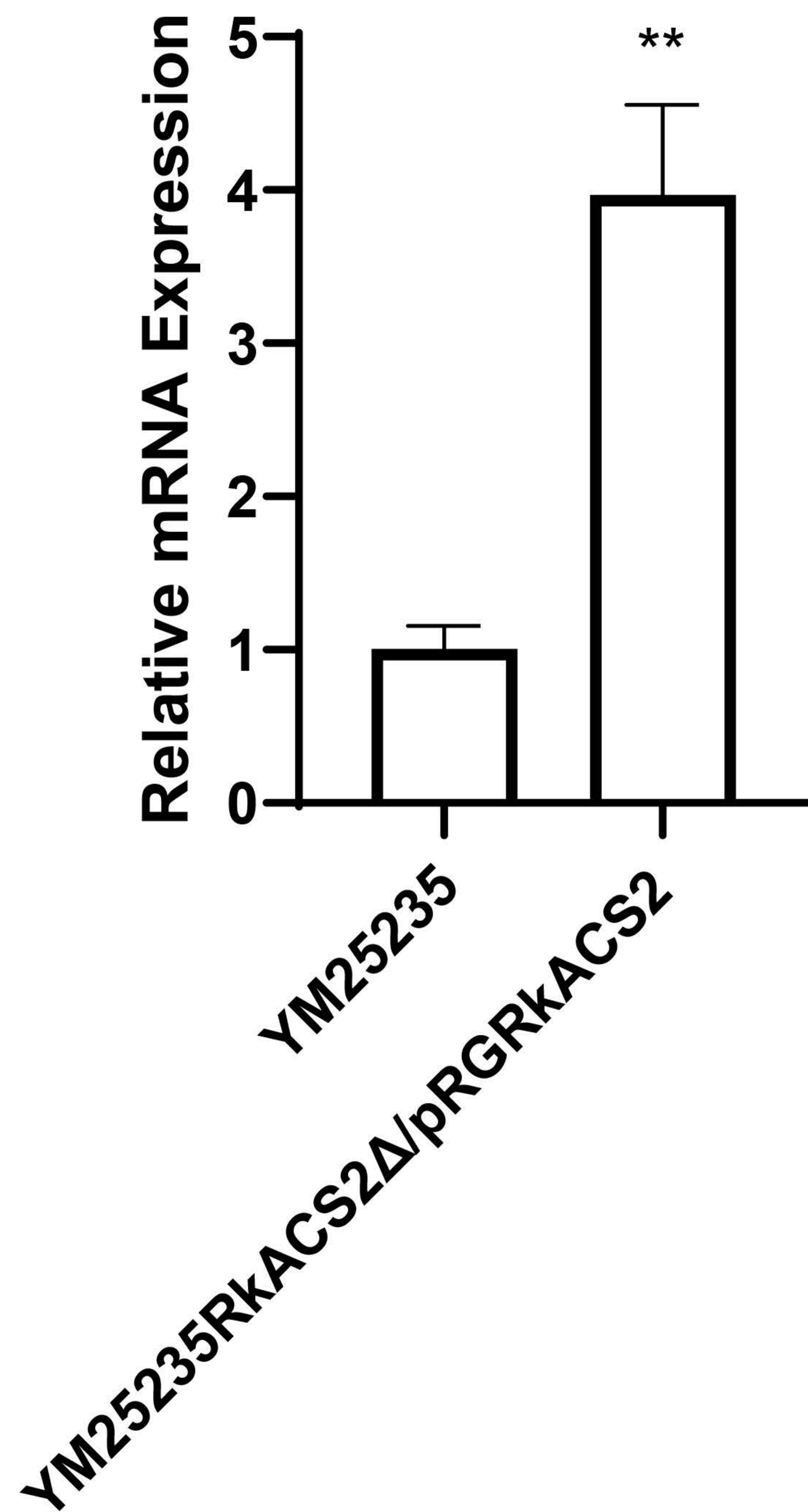

Supplement: Supplementary file 1 — Supplementary file1 (PDF 174 KB) [file 253_2025_13534_MOESM1_ESM.pdf]
