# Supplementary material for: Divergent roles of the acetyl-CoA synthetases RkACS1 and RkACS2 in carotenoid and lipid biosynthesis in Rhodosporidium kratochvilovae
Source: Appl Microbiol Biotechnol. 2025 Jun 7;109(1):140. doi: 10.1007/s00253-025-13534-x (PMC12145312; doi:10.1007/s00253-025-13534-x)
Supplement: Supplementary file 2 — Supplementary file2 (PDF 82 KB) [file 253_2025_13534_MOESM2_ESM.pdf]

Table S1. sgRNA design table.

	20-nt gRNA (NGG) (5'-3')	gRNA position	Edited gene
SgRNA1	GTCTCCTGCCTCGAAAGAGC(CGG)	-799 to -780	*RkACS1*
SgRNA2	CTGCCAGTACGTGTCGACGA(CGG)	-2189 to -2170	*RkACS1*
SgRNA3	GAAGAGGTTGCAGATCTGTG(CGG)	-1960 to -1941	*RkACS2*
SgRNA4	GACGGCGTGCTCAACCCGGG(CGG)	+2481 to +2500	*RkACS2*

Table S2. Primer pairs for PCR validation

	Primer sequence (5'-3')	Primer position
RkACS1-F1	ATGACCGAACACACCTACGAC	+1 to +21
RkACS1-R1	CTACGCCTTGGCGAACTT	-3055 to -3038
RkACS1-R2	CAGTACCACTCCCAAGCCTC	-2140 to -2121
RkACS2-F1	ATGGCCCCGACGTCTAC	+1 to +17
RkACS2-R1	CTACAGCTTCGCCTTGACG	-3036 to -3018
RkACS2-R2	CCTGAAGGTCCATCCCCAAC	-2033 to -2014

Table S3. Primer pairs used for quantitative real-time PCR.

Gene	Primer sequence (5'-3')	
SSU rRNA	F: CCATTCACTTACAAACACAA	R: CACCACCAGATTCACTAA
ACS1	F: CGTCGAGCTGTACAAGGAGT	R: AACCACTGTACGTCTCCTGC
ACS2	F: ACCTCGATGCGTTCTGGA	R: AGCATGTTCTCGGCGTAGTT
HMGCR	F: CATTGTCACCGTCTTCTG	R: GTCGTCGTAGAGGAGGAA
IPI	F: GACTTTGCCTACCTTACC	R: TGAGGAAGAGGATGTAGT
CrtYB	F: GTTCTTCTTGTGGGAGTG	R: CCGCTTCTTCAATCTCAA
CrtI	F: GAGAAGGGTTTCGAGGGCTT	R: ATGGAGAGGAGCGAGGTGAA
FAS1	F: ACACTACAACGAGAAGGCCG	R: CGCTGGTTGATGTAGAGGCA
ACCS	F: GCACGACCGAGTTCCTCTAC	R: GTTGACGCCCGAAACCATTT
ACOX2	F: ATTCACGACCTCACCAAGGC	R: CACTCCATATCGCGTCCAGA
ACAA1	F: GTCGAGTCGATGACCCAAGG	R: ACGTTCTCGCTCGTGATACC
